# Assessing communities of practice in health policy: a conceptual framework as a first step towards empirical research

**DOI:** 10.1186/1478-4505-11-39

**Published:** 2013-10-20

**Authors:** Maria Paola Bertone, Bruno Meessen, Guy Clarysse, David Hercot, Allison Kelley, Yamba Kafando, Isabelle Lange, Jérôme Pfaffmann, Valéry Ridde, Isidore Sieleunou, Sophie Witter

**Affiliations:** 1 Department of Public Health, Institute of Tropical Medicine (ITM), Nationalestraat 155, 2000, Antwerp, Belgium; 2 Facilitator of one of the Harmonization for Health in Africa Communities of Practice; 3 UNICEF West and Central Africa Regional Office (WCARO)BP 29720, Dakar-Yoff, Senegal; 4 Département de Santé Publique et de Biologie Médicale, Institut de Recherche en Sciences de la Santé (IRSS)03 BP 7192, Ouagadougou, Burkina Faso; 5 Maternal and Neonatal Health Group, Faculty of Epidemiology and Population Health, London School of Hygiene and Tropical Medicine, Keppel Street, London WC1E 7HT, UK; 6 Research Centre of the University of Montreal Hospital Centre (CRCHUM)Tour Saint-Antoine, 850 rue Saint-Denis, Montréal H2X 0A9, Canada; 7 FEMHealth project coordinator, Immpact, University of Aberdeen, 2nd Floor, Health Sciences Building, Foresterhill, Aberdeen AB25 2ZD, UK

**Keywords:** Communities of practice, Evaluation, Health policy, Knowledge management, Knowledge translation

## Abstract

Communities of Practice (CoPs) are groups of people that interact regularly to deepen their knowledge on a specific topic. Thanks to information and communication technologies, CoPs can involve experts distributed across countries and adopt a ‘transnational’ membership. This has allowed the strategy to be applied to domains of knowledge such as health policy with a global perspective. CoPs represent a potentially valuable tool for producing and sharing explicit knowledge, as well as tacit knowledge and implementation practices. They may also be effective in creating links among the different ‘knowledge holders’ contributing to health policy (e.g., researchers, policymakers, technical assistants, practitioners, etc.).

CoPs in global health are growing in number and activities. As a result, there is an increasing need to document their progress and evaluate their effectiveness. This paper represents a first step towards such empirical research as it aims to provide a conceptual framework for the analysis and assessment of transnational CoPs in health policy.

The framework is developed based on the findings of a literature review as well as on our experience, and reflects the specific features and challenges of transnational CoPs in health policy. It organizes the key elements of CoPs into a logical flow that links available resources and the capacity to mobilize them, with knowledge management activities and the expansion of knowledge, with changes in policy and practice and, ultimately, with an improvement in health outcomes. Additionally, the paper addresses the challenges in the operationalization and empirical application of the framework.

## Background

Proactive management of knowledge is today seen as a key strategy to ensure the performance and success of organizations or systems. This is true also in the health sector [1,2], where, over the last decade, health system researchers have paid more attention mechanisms to ensure better sharing of knowledge, with a particular focus on the challenge of getting evidence into policy and practice. In parallel, information and communication technologies have experienced tremendous developments, allowing knowledge management processes (in terms of storing, retrieving, and sharing knowledge, in particular) that were unimaginable a few decades ago.

These changes have led to the emergence of new strategies of knowledge management in global health.^a^ Most of the time, they tap into the power of online technologies (emails, listservs, websites, blogs, social media, etc.) to enhance connections between experts, but many also care about cultivating a certain degree of face-to-face interactions. Developing and implementing such strategies may require a substantial commitment of resources from sponsors, members, and facilitators. The question of their effectiveness and efficiency, among other dimensions, is therefore an important one.

This paper focuses on a specific knowledge management strategy: the community of practice (CoP). Our main objective is to contribute to the emergence of a conceptual framework for understanding and assessing CoPs in health policy. This step also allows the delineation of a research agenda for the empirical application of the proposed framework.^b^

The paper is organized as follows. First, the concept of CoP will be introduced. We will make a distinction between ‘de facto’ CoPs and those set up as explicit knowledge management strategies; the focus of the paper will be on the second type. After this general introduction to the strategy, we will provide some background information on transnational CoPs in health policy and highlight some of their characteristics. The development of the framework has been preceded by an intensive exploration of the literature, which we report on in our methods section. We then present our framework; a discussion follows that addresses issues regarding the ‘operationalization’ of the framework (i.e., a reflection on the methodological challenges that the empirical application would involve). Finally, an agenda for further empirical research is proposed.

### The emergence of the concept of communities of practice

We owe the concept of CoP to anthropologists who revealed, through grounded, detailed empirical work, the situated character of practical learning [3]. From their observations across cultures and situations, they identified mechanisms and principles contributing to an effective transfer of practical knowledge. The recognition that practical learning takes place mainly through social interactions and in settings as close as possible to those of the actual practice [3,4], led them to label the overall mechanism as a ‘community of practice’.

Wenger and colleagues defined a CoP as “a group of people who share a concern, set of problems, or a passion about a topic, and who deepen their knowledge and expertise in this area by interacting on an ongoing basis” [5]. A first stage in their work was the observation that CoPs spontaneously emerged in various professional sectors, as they are particularly effective in situations where it is important to ensure the transfer of tacit knowledge into practice (compared to, for instance, the key contribution of the internship in medical education). A second stage was their recognition that the CoP model could be theorized, formalized and made instrumental for application in a more purposeful way by individuals or organizations with the explicit aim of improving knowledge management towards predefined objectives. The potential of the strategy as a knowledge management tool to foster professional development, create and share knowledge across units, departments or branches was rapidly identified in the business sector. In comparison to other knowledge management strategies, its strength indeed lies in its promotion of an environment conducive to learning and exchange by fostering social relationships and recognizing the importance of both implicit and explicit knowledge, emphasizing interactions in a climate of mutual trust [5]. This quality was later recognized in other sectors, including education and, recently, health [6].

### ‘De facto’ and ‘instrumental’ communities of practice: two tracks for analysis

The concept and its managerial development attracted the attention of numerous researchers, which has led to a growing body of literature that documents and reviews the experiences of those participating in CoPs, including in the healthcare sector (e.g., [7-10]). Two systematic reviews have been produced [11,12]. As we report in our methods section, this literature is vast, ambiguous and conceptually diffuse. We believe it is important to make a distinction between two angles in the analytical approaches, which actually reflect the process through which the concept emerged.

Some scholars adopt a definition of CoP that stresses the need for a shared *domain* of expertise and repertoire of practices, as well as the existence of a sense of *community* and of interactions that are *meaningful* and that consolidate the practitioner *identity*[5]. Equipped with this list of key attributes, the researchers then analyse any interactive groups (e.g., online groups) to check whether they fulfil the criteria and can therefore be identified as CoPs (e.g., [8]). This view has a strength: it not only proposes an analytical grid to describe a pre-existing social arrangement [10], it also offers a theoretical proposition of what is required for the transfer of tacit knowledge to succeed.^c^

The second view deals less with scientific validation criteria; it embraces the broader agenda of knowledge management (often referred to as K* in the literature) and observes the functioning and effectiveness of knowledge management strategies which explicitly identify themselves as CoPs. In this body of managerial literature, the focus is often on highlighting ‘good practices’, especially in terms of stewardship and facilitation.

This paper adopts the second view: the instrumental, managerial approach, which considers CoPs that are purposefully set up as a strategy to manage knowledge and often aim toward pre-defined objectives beyond mere knowledge management. In order to avoid misunderstanding, we will use the acronym CoP^KM^ to refer to these communities of practice set up as knowledge management strategies and which may not fulfil all the attributes mentioned above (for instance, the starting point of some CoP^KM^ may be quite far from a ‘shared’ identity). The case for adopting an instrumental approach is strong; as reported in the section presenting the framework, we adhere to a view of organizations as collective arrangements that individuals set up, join or support with the aim of achieving individual and collective goals [13]. The fact that these goals remain implicit (e.g., because of a lack of centralized stewardship, as it may be the case with some de facto CoPs) or are made explicit (e.g., by the facilitator in charge of a CoP^KM^) does not change the fact that CoPs are artefacts. They are adopted because they serve functions; therefore, when one studies a CoP, the ultimate question is not one of ascertaining its compliance with a definition^d^, but one of evaluating whether it is, as a collective arrangement, superior to alternative collective arrangements, with respect to collective and individual goals.

Focusing on answering this latter question also allows for a more detailed evaluation agenda. In particular, evaluations could aim to describe the collective arrangement; assess its efficiency and effectiveness as a knowledge management tool, but also as a strategy to reach broader objectives (such as contributing to better performance and improvements in policy and practice); and identify factors that could explain its effectiveness (or not) and derive from that observation more generic lessons for other CoP^KM^.

The recognition that any collective arrangement is instrumental also shows that the two views of CoPs are not mutually exclusive. It could be argued that efforts to foster a CoP^KM^ may contribute to the actual emergence of a ‘de facto’ CoP or, conversely, that facilitators of a CoP^KM^ will have an easier task in achieving their objectives if they can build on the prior existence of a ‘de facto’ CoP. Accordingly, as we shall see, one dimension of our framework incorporates key attributes of ‘de facto’ CoPs, as these features are susceptible to enhancing knowledge management.

Concisely, our focus on the managerial and instrumental aspect has practical value towards our goal, which is to improve effectiveness of CoP^KM^, including those in which we are involved. This goal requires developing a capacity to monitor and evaluate, which itself requires a more explicit ‘theory of change’, for the CoP^KM^ as a whole, but also for specific activities. We believe that a preliminary step in this ambitious endeavour is to map and organize the dimensions which matter. This is the main purpose of this paper, paying particular attention to transnational CoPs in health policy.

### Transnational communities of practice in health policy

The development of this framework arose due to some very specific needs: ours. As knowledge experts (mainly in healthcare financing), together with different actors, we have launched or are playing a supportive role in several CoP^KM^.

Our own efforts are part of a larger movement in global health: in recent years, several actors have adopted the CoP^KM^ as a strategy to enhance exchange and co-production of knowledge across countries. Their domains of interest are various (see endnote^a^ and the illustration of a transnational CoP^KM^ in health policy: the performance-based financing CoP section below), but often relate to ‘health policy’, which, according to the WHO [14], “refers to decisions, plans, and actions that are undertaken to achieve specific health care goals within a society”. Besides, their membership is often ‘transnational’, i.e., distributed across continents and languages. Efforts to map the existing transnational CoP^KM^ in health policy have yet to be undertaken. However, based on our experience, we suggest that transnational CoP^KM^ in health policy have specific characteristics.

First, a key concern of facilitators and patrons of CoP^KM^ in health policy will often be to ensure that the activities are in line with a wider process of mobilization of local, national and international resources for the achievement of the health care goals. Obviously, the patrons of a specific CoP^KM^ may decide to narrow its contribution to a particular issue in the health policy chain (e.g., how to do research on health policy) and focus its recruitment on one category of knowledge holders (e.g., researchers). However, some CoP^KM^ have also been set up with broader objectives, such as directly influencing the content of health policy. Such a CoP^KM^ may then make the conscious choice of recruiting its members among different categories of actors, especially all those who hold knowledge relevant for progressing towards the health policy goals (or at least those that are favoured by the patrons). This ambition may require reaching out across professional groups (clinicians, managers, analysts, etc.), academic disciplines (medicine, economics, political sciences, etc.), organizations [15], hierarchies and countries, in other words, across different ‘regimes’ of knowledge holders [9,16]. In fact, some of us have argued that the CoP^KM^ strategy, due to its inclusiveness, could be particularly apt at bringing different types of knowledge holders onto the same platform, especially when the focus is on implementation issues [17]. However, such an ambition raises particular challenges, and certainly reduces the chance of building a de facto CoP, as members do not share the same repertoire of practices.

Secondly, knowledge in health policy is more context-specific than in other domains (compared to, for example, a community of software programmers or even clinical staff [8]), because decisions on policies and on their implementation are not only based on technical issues, but also on political and cultural considerations and depend essentially on interactions between institutional actors and contextual factors [18,19]. This influences the nature of the practices of CoP^KM^ in health policy (focused on identifying problems, assessing possible solutions, designing, budgeting, monitoring and evaluating schemes, developing skills for system analysis, considering political sensitivity, etc.), the type of knowledge shared (e.g., less focus on practical tips) and the way it is shared (e.g., through promotion of expert mobility across countries).

Finally, transnational CoP^KM^ in health policy are, by definition, widely distributed, extending across countries, and sometimes continents and languages. For this reason, they may often take the form of virtual CoP^KM^, taking advantage of information and communication technologies (ICT), even if many arrange for some face-to-face interactions or cross-country professional mobility [5,20]. Given these characteristics, transnational CoP^KM^ in health policy face specific challenges that influence their creation, development and impact.

Because of our own needs, our framework is intended to capture these additional challenges. However, as it also addresses many of the challenges common to most CoP^KM^, it may have relevance for other situations, even outside the health sector.

### An illustration of a transnational CoP^KM^ in health policy: the performance-based financing CoP [17]

#### Domain of knowledge

Performance-based financing (PBF) is a health care financing strategy stressing the role of incentives in the public health sector in low-income countries [21]. This strategy is receiving increased attention from governments and donors, especially in sub-Saharan Africa. As a result, there is a strong demand for knowledge production and sharing in this domain, both at country and regional level.

#### Objective of the CoP^KM^

The main aim of the PBF CoP is to build a critical mass of high-quality African experts in PBF. The best option to do so is to strengthen the capacity of practitioners already involved in implementing PBF schemes and enhance the sharing of their expertise at regional level. The CoP^KM^ also aims at consolidating the body of knowledge on PBF through the identification and dissemination of good practices. The role of some pioneer countries is critical in the production and promotion of approaches that proved to work. Allowing for the transfer of good practices, while at the same time securing enough attention and openness to constraints and opportunities specific to each context, still remains a challenge.

#### Process

The PBF CoP was launched in Burundi in February 2010. The majority of the participants at the launching event were African experts with substantial experience in designing, implementing or assessing PBF schemes. Subsequently, an online discussion group was launched (http://groups.google.com/group/performance-based-financing). To date, the group gathers around 1,100 experts, active in different sections of the knowledge chain. They are based in many regions of the world, but predominantly in Africa, including in settings where access to internet is a challenge. Different knowledge activities are organized by the CoP^KM^: workshops, a collective book, a working paper series, a toolkit, a blog, e-discussions, and so on. The PBF CoP is supported by different sponsors, including aid agencies, consulting companies, international NGOs and research institutes. It has two part-time facilitators.

#### Assessment

PBF is expanding rapidly in sub-Saharan Africa; while the PBF CoP’s own contribution is difficult to ascertain, it has established itself as the main platform for knowledge exchange and development on PBF. Some early analyses of the discussions on the online forum have confirmed the focus on a specific policy domain, the collective sharing of a technical repertoire and the emergence of an identity and community spirit, all key features of a de facto CoP. The emergence of the de facto CoP has probably enhanced the completion of some knowledge activities, but is not enough to assure the success of all projects, especially the most ambitious ones.

## Methods

In order to develop the conceptual framework for analysis and assessment of transnational CoP^KM^ in health policy, an exploratory review of the existing literature was conducted. The approach adopted was that of a broad scoping study. Criteria for inclusion were not based on a pre-defined list nor on the quality of the studies, but on their relevance to our research question, which was defined *post hoc,* once authors were more familiar with the body of literature [22,23]. Initially, PubMed and Google Scholar databases were searched by using key words, such as ‘systematic reviews’, ‘evaluation’, ‘assessment’, ‘monitoring’, ‘value creation’, ‘framework’, ‘success factor’, ‘limitations’, as referred to CoPs within and outside the health sector. We then adopted a snowball technique to identify further documents in the published and grey literature, and further searched online archives and discussions of existing CoPs, in particular in the health domain. Because the existing literature is so vast and diverse (e.g., ‘communities of practice’ totalized 2,780,000 counts in Google Scholar in August 2013) and not always applicable to the case of global health policy, the document search was not systematic nor exhaustive. Its main aim remained instrumental in providing a background and overview of the previous work in this subject and therefore it was carried out up to the point where the authors deemed that all elements relevant for transnational CoP^KM^ in health policy were included.

Undertaking such a literature search allowed us to identify and reflect on some key issues and elements relevant for the understanding and assessment of CoP^KM^ in health policy. We then combined this with our own experience and insights on transnational CoP^KM^ in health policy to build a conceptual framework. To corroborate the findings, a consultation process was also undertaken [22]. A first sketch of the conceptual framework was presented and discussed at two meetings organized respectively in Antwerp, Belgium (on August 31, 2011) and in Bamako, Mali (on November 20, 2011). These meetings gathered facilitators and members of one or more transnational CoP^KM^, who commented on the draft document. Their experience and expertise provided critical inputs to refine the conceptual framework. The future use of this conceptual framework for the empirical assessment of CoP^KM^ will provide a further opportunity for testing, refining and validating it.

## Findings

### Assessing CoP^KM^: key elements from the literature

The literature review helps identify some critical elements relevant for the analysis and assessment of CoP^KM^ in health policy to build upon for the construction of our conceptual framework. The 25 key papers retained are listed in Table 1. Most of the papers provide theoretically interesting and rich ideas, but, with few exceptions [24,25], none of the documents presents an empirical application of an evaluation framework, highlighting a gap: despite the wide theoretical and practical interest on CoPs, there is a lack of evidence-based propositions for their evaluation.

**Table 1:** Key documents identified by the literature review, focusing on elements and propositions relevant for the assessment of transnational CoPs in health policy

**Authors**	**Year of publication**	**Reference**	**Sector of reference**	**Main dimension of focus**	**Key elements, findings and propositions**
	**FRAMEWORKS FOR ASSESSING CoPs’ PERFORMANCE**				
Schenkel et al.	2000	[26]	Management	Performance of CoPs	Social network analysis.
McDermott	2002	[27]	Business	Return On Investment (ROI)	Pyramidal framework, which starts from ‘activities’ at the base and moves upwards to ‘outputs’, ‘value’ and ‘business results’.
Wenger et al.	2002	[5]	Business/Management	ROI	Simple method for calculating an approximate ROI value.
Arora et al.	2002	[28]	Business	Performance of CoPs/ROI	Balanced scorecards.
Millen & Fontaine	2003	[29]	Business	Performance of CoPs/ROI	Causal model for community interactions and benefits, which categorizes benefits into ‘individual/personal’, ‘community’ and ‘organizational’.
Lee et al.	2005	[30]	Business/Management	KM performance/ROI	Complex, formalized method to calculate ROI for KM activities.
Helms et al.	2007	[31]	Management	Performance of CoPs/ROI	Knowledge network analysis.
Scarso et al.	2009	[24]	Business/Management	Success factors/ROI	Identifies two external influences (the organization’s own knowledge strategy and the context) and four internal/constitution characteristics of CoPs (organizational, cognitive, economic and technological dimensions) to explain the CoPs success (applied to the case of a multinational oil company).
Braithwaite et al.	2009	[32]	Health	Performance of CoPs	Protocol presenting a methodology for the “development, design, testing, refinement, simulation and application of an evaluation framework for communities of practice and social-professional networks”.
Wenger et al.	2011	[33]	Education	Assessing ‘value creation’	A very detailed, comprehensive guide for promoting and assessing ‘value creation’ (a performance measure of the level of learning enabled) for CoPs and networks in the education sector. Includes a conceptual framework and practical methods and tools.
Ranmuthugala et al.	2011	[34]	Health	Performance/role of CoPs	A second study protocol (following Braithwaite et al. 2009) proposing ‘realist evaluation’ combined with ‘social network analysis’ as a tool for the development of such a framework. Both protocols focus mainly on the application of CoPs to healthcare activities, specifically in Australia.
ADB	2011	[35]	Development	Evaluation of KM strategies	Use of the DAC Criteria for Evaluating Development Assistance as a tool for assessing KM strategies. The DAC criteria are relevance, effectiveness, efficacy, sustainability and impact.
	**LIMITATIONS OF CoPS**				
LeBaron	2000	[36]	Education	Limitation of CoPs	Cultural and social values of collaboration vs. individual success.
Yanow	2004	[37]	Management	Limitations of CoPs/Hierarchies	Role of local vs. expert knowledge; distinction between horizontal, geographic periphery and a vertical, hierarchical periphery.
Roberts	2006	[38]	Management	Limitation of CoPs	Power structures and hierarchies; time needed to evolve and mature; resistance to change.
Kerno	2008	[39]	Management	Limitation of CoPs	Time constraints; organizational hierarchies; regional culture.
	**ELEMENTS FOR THE SUCCESS OF CoPs**				
Johnson	2001	[40]	Education	Early research on virtual CoPs’ characteristics	Different levels of expertise; fluidity of knowledge flows (vs. withdrawal/attrition); community knowledge greater than individual knowledge; environment of safety and trust.
Sveiby & Simons	2002	[41]	Management	Trust	Collaborative climate is one of the major factors influencing effectiveness of knowledge management.
Levin et al.	2004	[42]	Management	Trust	Trust as essential for knowledge sharing.
Wenger et al.	2005	[43]	Management	Use of ICT	Contribution of technologies to CoPs; new tools and challenges in the use of ICT; description of technologies the CoPs use to “create a sense of togetherness over time and across distances”.
Bourhis et al.	2005	[25]	Management	Leadership	Role of the community leaders and coach to respond to challenges in a way adapted to the CoP characteristics (presenting empirical case studies).
Cargill	2006	[44]	Management	Leadership	Role of leaders and leadership issues.
Ardichvili et al.	2006	[45]	Management	Culture	Cultural influences and potential cultural barriers in knowledge sharing and participation.
Usoro et al.	2007	[46]	Management	Trust	Trust as predictor of knowledge sharing behaviours. Trust is analysed across three dimensions: perceived competence, integrity and benevolence of the CoP.
Kraut & Resnick	2011	[47]	Management	Use of ICT	Possible designs improving the success of online communities.

### Proposed framework to assess transnational CoP^KM^ in health policy

**Figure 1:**
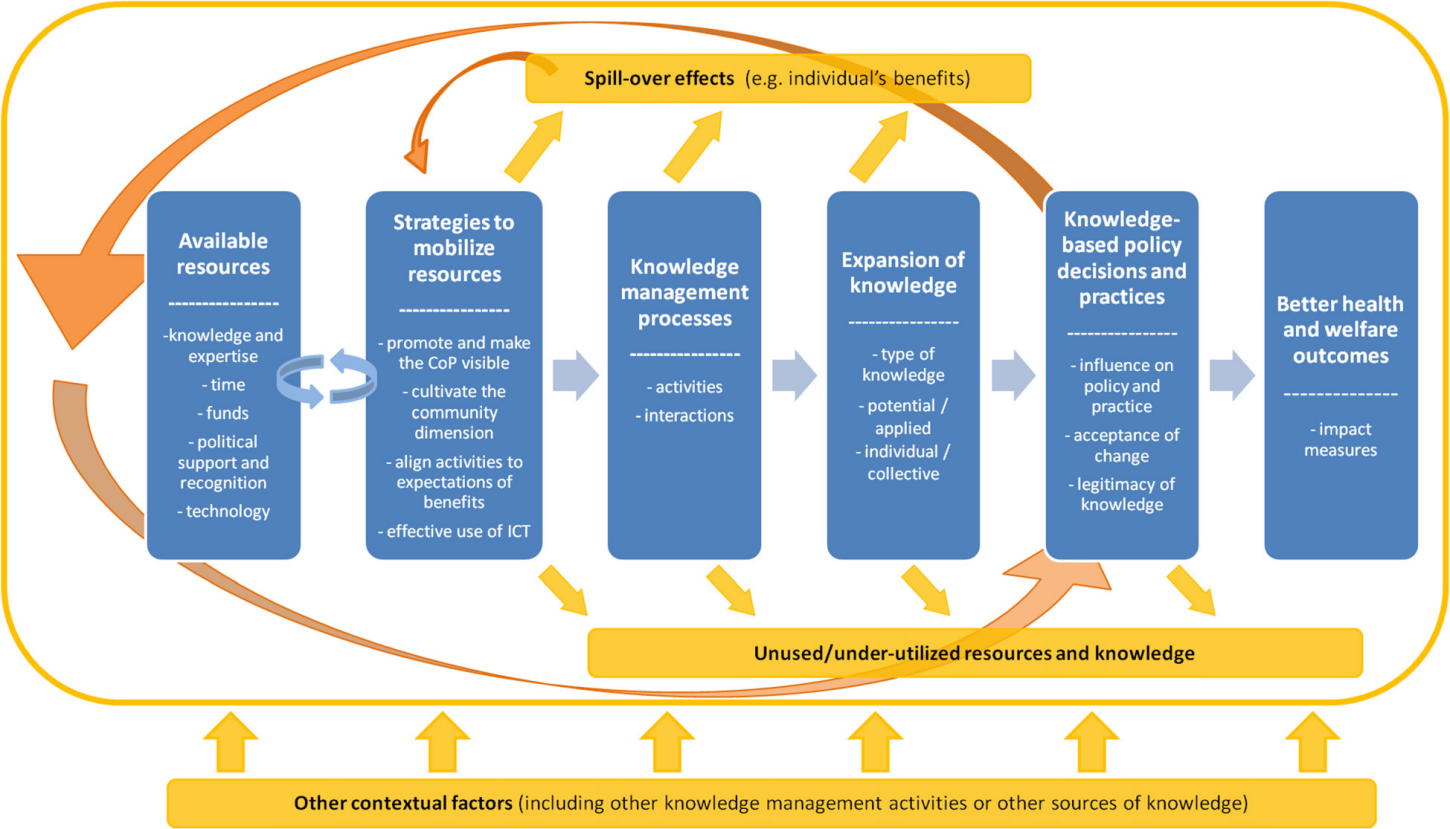
A simplified graphic representation of the conceptual framework for assessing communities of practice in health policy.

In order to build our conceptual framework, we undertook an organic revision of the main points emerging from the literature, integrating them with our experience and propositions. This step allowed for the identification of a series of elements central to the understanding and analysis of the CoP^KM^ in which we are interested. We then reorganized these elements into six ‘dimensions’ in a way that reflects a simplified representation of a CoP^KM^ and its functioning, from a managerial, instrumental approach, referencing and taking into account the specific features and challenges of transnational CoP^KM^ in health policy (Figure 1).

Grounded in the ‘instrumental’ perspective on CoPs, the framework retraces and analyses the role that knowledge (produced, created and managed through the CoP^KM^) plays in the process of selecting and implementing health policies, which, in turn, may affect health outcomes valued by citizens. In this sense, the framework is normative as it entails that the CoP^KM^ produces knowledge relevant and valid in reference with regard to the health system goals, as defined by the WHO [48]. Although the framework recognizes many non-linear loops, spill-over effects, etc., because of its focus on the instrumental role of CoP^KM^, it mainly draws and focuses on the ‘input-process-output-outcome’ logic that is familiar to public health experts [49] or the theory-based evaluation approach [50]. Following that logic, it retraces a path between a series of dimensions that facilitators and members activate to ensure the CoP^KM^ delivers results. However, contrary to analytical models of process in public health, and in order to allow consideration of the complex dynamics and processes involved, under this framework, resources are not assumed given. Instead, the challenge of the CoP^KM^ is to constantly and dynamically mobilize new resources for its development and success.

The conceptual framework also presents links with institutional and organizational theories [13]. It endorses a view of the CoP as an organizational modality that individuals set up, join and support with the aim of achieving individual and collective, implicit or explicit goals. The individual goals relate to gaining knowledge, as well as other benefits such as visibility, social capital, influence or even business opportunities. The collective goals – especially relevant to patrons – include knowledge objectives, but also others, perhaps less explicitly, such as influencing policy processes and policy decisions. Under the proposed framework, the hypothesis is that, in order to do so, the CoP^KM^ patrons and facilitators mobilize critical resources (knowledge and expertise, time, funds, political support and technologies) through governance rules and processes fostering voluntary human interactions. Although the framework portrays a managerial vision of knowledge production and management, it is not unaware of the numerous and varied reasons that contribute to the decision of supporting a CoP^KM^, leading it, participating in online discussions, etc. Indeed, as also highlighted by standard organization theories (e.g. [51]), a CoP^KM^ not generating value for its members in an efficient manner loses support and may even disappear.

Each of the dimensions of the framework (represented as boxes in Figure 1) is further described in the following paragraphs.

### Available resources

Certain critical resources are at the base of the functioning and the effectiveness of a CoP^KM^, and are provided mainly by the CoP^KM^ members and by its patrons. Those resources belong to different categories:

•*Knowledge resources* include different types of knowledge and expertise held by the members. They also include access to information (such as scientific journals) for the CoP^KM^, collectively and through its members, and any pre-existing knowledge-sharing platforms.

•*Time resources* relate to the time that members choose to allocate to the CoP^KM^ activities and the time that their organizations allow them to take out of other, more formal activities.

•*Financial and other material resources* include funds and in-kind allowances (human resources, meeting space, web space, materials, etc.).

•*Political resources* refer to the buy-in of key organizations in the domain of practice of the CoP^KM^ and include the public recognition and reputation of the community.

The correct use of *technological resources* is critical for the performance of widely distributed, transnational CoP^KM^. ICT plays a key role in connecting geographically dispersed members to create a sense of ‘togetherness’, as well as providing them with a platform to share, store, and access the explicit and implicit knowledge of the community [43].

### Strategies to mobilize resources

A well performing CoP^KM^ is able to implement strategies to successfully mobilize both available and new resources and to increase them over time.

The literature and our experience suggest that the core group of facilitators of the community plays a critical role and is instrumental in facilitating resource mobilization [25,44]. This core group is responsible for four main sets of tasks:

1. Clarifying the domain of focus, defining the strategic objectives of the CoP^KM^, ensuring that enough focus is kept on the repertoire of practices, promoting and making the CoP^KM^ visible and carrying out public appraisals and (self-) assessments of the community. This is fundamental to *mobilizing financial and political resources* and to ensure the evolution of the CoP^KM^ and its sustainability in the long-term.

2. Cultivating the community dimension of the CoP^KM^, going across knowledge ‘regimes’ (if there is any pluralism at this level) and creating an environment that is conducive to knowledge exchange. This is critical to *mobilizing knowledge and time* resources by increasing the active participation of members. To achieve this, some elements should be taken into consideration and are critical to explore:

– The *power structure* of the community. While facilitators attempt to foster ‘horizontal’ CoPs in order to mitigate external, pre-existing hierarchies among members and ensure wide participation, it is unavoidable that some existing hierarchical features will persist and others may emerge internal to the CoP^KM^ (see the growing literature on different roles – the ‘lurker’, the ‘novice’, the ‘elder’, the ‘poster’ in online communities [52]). However, the fact that some members may remain in peripheral positions could reduce the effectiveness of the CoP^KM^[38].

– The *regulatory mechanisms* established by the CoP^KM^. These mechanisms aim to ensure smooth and relevant discussions, avoiding contributions that are inappropriate for their content (e.g., spam) or their form (e.g., interpersonal conflicts).

– The level of *trust.* Trust and a collaborative climate enable the sharing of knowledge, particularly of a tacit nature [38,41,42,46]. The ‘*fluidity*’ of the community, i.e., the ease with which information and knowledge are shared among members, as opposed to ‘withdrawing’ or attrition [40]. Within this dimension, cultural differences and potential cultural or language barriers should be taken into account [45].

– The passion for the topic and the commitment and *ownership* of the members, are important to forge a common identity of the community [5]. As highlighted by early work on CoPs, these features foster a positive environment and good relationships enabling explicit and tacit knowledge to flow within the community.

3. Aligning CoP^KM^’s activities and products to individual and organizational expectations of benefit. If individuals and organizations have (intrinsic or extrinsic) reasons to participate actively in the CoP^KM^ (or to allow participation), more *knowledge and time resources* will be mobilized. The framework allows capturing the determinants of the motivation to participate under the different dimensions. For example, do people participate because of the interactions (networking), the expansion of knowledge (learning), to increase their social capital or because they care about improving policy making and contributing to better health outcomes?

4. Choosing and adopting the relevant *ICT*, including the platform design features that are appealing, enable the socialization of new members and encourage commitment and appropriate contribution by members in a cost-effective manner [47].

Obviously, these first two dimensions (available resources and strategies to mobilize them) are closely related, which makes it almost impossible to identify their causal and chronological relationship, so that they should be looked at jointly. Indeed, mobilizing new resources also means that more will be available for the CoP^KM^. For this reason, from the beginning and continuously throughout the CoP^KM^ life, a sort of virtuous cycle of mobilization of new and existing resources should be in place.

### Knowledge management processes

Once resources are mobilized and available, they are used to foster knowledge management processes, which include knowledge creation, identification, storage, share and use [53]. Therefore, this dimension aims to capture the reality and nature of the knowledge processes realized by the active members. Knowledge management processes materialize in the *activities* that the CoP^KM^ organizes and performs (workshops, online discussions, formal meetings, websites, etc.), as well as in the *interactions* that it fosters among its members (web posts, collective or private emails, formal and informal discussions, and so on). As a CoP^KM^ is not focused only on the quantity of activities and interactions promoted, it would be important to assess also their quality and their relevance to i) individual members, ii) their organizations, and iii) the CoP^KM^’s objectives and aims. A key question is whether activities focus on improving the repertoire of practices.

### Expansion of knowledge

Knowledge management processes aim to bring about an expansion of knowledge. The knowledge produced has different characteristics:

•It can be of *different types*: explicit or implicit; theoretical, statutory or applied; based on scientific evidence, on field experience or on experts’ opinions; specific to one regime or accepted between different regimes; a matter of debate or consensus within the CoP^KM^ and outside, etc.

•Knowledge can be *potential or applied*[33]. The first refers to knowledge whose potential value could be realized later and is stored in the form of knowledge capital, which includes skills (human capital), relationships (social capital), access to resources (tangible capital) and reputational capital. In contrast, applied knowledge is fully realized and produces changes in individual practices.

•The expansion of knowledge can be realized at the *collective or individual level.* In this latter case, it is interesting to understand who benefited from the expansion of the knowledge, i.e., whether it is only the members, or some of them (and if so, whom), or if the expansion had spillover effects to a wider audience. This analysis would highlight important distributional and equity issues within and beyond the CoP^KM^.

Each community may focus on different knowledge characteristics among those described above, according to its domain of interest, repertoires of practices and specific goals (for example, some CoP^KM^ are focused on production and synthesis of evidence-based knowledge, while others aim to share implicit ‘know-how’ among individuals or even different actors), and based on the individual and organizational benefits that its members expect.

### More knowledge-based policies and practices

For the patrons of a health policy CoP^KM^, a key objective will often be to ensure that policy decisions and implementation practices have a sounder knowledge base than is usually the case (see [17] for an example from health care financing policies in low-income countries). The achievement of this objective would probably consolidate the legitimacy of the CoP^KM^ and therefore support it, both internally and externally. However, although one would expect scientific evidence to be a central component of the knowledge shared among members, evidence in the health policy domain often remains partial as it cannot cover all possible policy situations and options; furthermore, it is very context specific [54]. This means that, on the knowledge to policy guidance path, some areas of uncertainty may be filled with the ideologies and societal and political preferences of members. Global health policy is a rather open arena; this creates some checks and balances for the CoP^KM^ (e.g., concern for attracting the support of other influential actors in the policy process will discipline the CoP^KM^); yet, it may not be enough. The shallower the evidence base, the greater the responsibility of the facilitators to protect dissident opinions, to organize eye opening activities and to practice self-assessment.

The transformation of knowledge into policy requires its acceptance by policy makers and implementers. This may occur if policy makers and implementers are active CoP^KM^ members themselves, or they consider CoP^KM^ members and/or their knowledge products as trustworthy, or if members (e.g., researchers, consultants), empowered by their enhanced knowledge or expert identity, contribute more effectively to the national policy process. Some CoP^KM^ in health policy are particularly attentive to gathering different knowledge holders and stakeholders on a common platform [17].

The CoP^KM^’s ability to produce authoritative policy recommendations or have them (or the underlying frameworks) internalized by its members will depend on its domain of knowledge, membership, facilitation and internal cohesion as well as on its ultimate objectives. A homogenous CoP^KM^ focusing on a narrow domain and aimed at promoting a particular view on it can produce clear policy messages, with the risk of the CoP^KM^ or its members overestimating the external validity of the related knowledge. Conversely, a CoP^KM^ focusing on broader issues and with a heterogeneous membership in terms of societal preferences may remain relatively open to possible options, but possibly at the cost of the capability to produce recommendations.

To avoid the possible risks involved in the prescriptive step towards policy recommendations, the CoP^KM^ must practice self-reflection and be attentive to two issues in particular. One issue is the *status of the knowledge* created. How can one combine consensus among members with rigorous demonstration? As mentioned above, evidence is often incomplete. The risk is that opinions (put forward by dominant members or by the majority of members) are accepted as validated evidence. Facilitators and members must beware of this and play an active role in identifying opinions as rather being hypotheses to be tested. Another potential pitfall for any CoP is that of ‘becoming a sect’ or a *static community*, not accepting change, and resistant to different developments in knowledge [5,38]. This risk may occur both during the translation of knowledge into policy and practice, but also earlier in the process when the CoP^KM^ (or its facilitation team) identifies issues to prioritize in terms of knowledge activities. At that stage, the CoP^KM^ may become dogmatic in its definition of relevant questions and ignore views challenging its ‘good practices’.

### Better health and welfare outcomes

Policy decisions and practices may lead to improved outcomes and reduced health inequalities. This is clearly the ultimate collective goal of several CoP^KM^ in health policy. Therefore, it would be ideal to be able to measure health and welfare outcomes, as well as the proportion of change attributable specifically to the CoP^KM^’s activities. However, this is highly complex and often not realistic. Firstly, it may take a long time for knowledge and expertise to finally result in better outcomes (because of delay in the uptake, e.g., the time needed to ban tobacco, and in the impact) and the time delay would make assessments difficult. Secondly, knowledge is just one element among numerous others in the production of good health: alone it cannot do much.

## Discussion

### Non-linearity of the conceptual framework

We have represented the role of CoP^KM^ in the process that leads to better policies and practices and better outcomes as a somewhat linear sequence. However, the process we are aiming to capture is obviously far more complex and often non-linear [55,56]. Many elements contribute to reinforce each other in a dynamic and iterative way. At the same time, along this process, the CoP^KM^ may generate secondary, spillover outcomes that go beyond the production and use of knowledge, such as creating solidarity amongst its members, reducing their isolation or offering career opportunities. The framework does not focus on these other outcomes, but this does not mean that they are negligible: in fact, they may be some actors’ main reasons for being involved with the CoP^KM^.

Additionally, the framework should not be read as a chronologically linear process. For instance, the resources that are available initially are not immutable for the CoP^KM^, but they interact dynamically and can be increased through the creation of virtuous cycles. As an example, public recognition is difficult to count on at an early stage, but could be successfully built over time. In the same way, at the beginning there may only be a small group of active members (or even just one ‘knowledge entrepreneur’). However, if the community develops in the right direction (e.g., by satisfying the benefits expectations of potential participants), it may be able to involve more members and add to the available knowledge capital. This argument points to an important feature of CoPs: they take time to evolve and mature. Therefore, to be effective, CoP^KM^ must be able to sustain their activities over time [38]. As a consequence, their assessment should include sustainability measures [57] and should not focus on applying the framework chronologically (i.e., look at available resources at an early stage, focus on the knowledge management activities during the maturity stage and evaluate the impact after the end of the CoP^KM^’s life), but should look dynamically at the different elements of the framework at regular intervals during the life of the CoP^KM^.

Finally, the framework should not be read as a causally linear process either. Indeed, the fact that there has been an expansion of knowledge does not automatically mean that policies will integrate such new knowledge [58]. The framework limits its aim to mapping a simplified path describing how an effective CoP^KM^ strategy could contribute to better health outcomes. Although it does not identify the causes that explain why impact was not achieved, it provides a series of dimensions to measure a possible progression towards better health outcomes. Briefly, while the framework does not provide a full theory of change for CoP^KM^, it is designed to be rich enough to capture the dimensions that matter and need to be thought through by actors committing resources to CoP^KM^.

### Applying the conceptual framework: choice of a methodological approach

The proposed framework is not prescriptive of a sole way of assessing CoP^KM^ by looking at all the dimensions proposed. Instead, it aims to provide a frame to organize, select and analyse different elements of CoP^KM^ and the dynamics between those elements. Thus, it must be tailored to the assessment needs of the evaluator, which will determine the research questions and the appropriate methods. The disciplinary methods adopted may vary; qualitative and quantitative methods are often to be used in conjunction. Indeed, numerous scholars argue that, in the assessment of CoPs, a focus on processes and outputs from the perspective of the community members and the use of “systematic anecdotal evidence” [5] are as important as quantitative evidence of impact [5,27,33,59-61]. The choice of the methodological tools will also depend on the dimension of focus. While, initially, the dimensions in the framework are mostly descriptive and the analysis can be performed using relatively simple quantitative indicators (e.g., study of the ‘demographics’ of the CoP or internet statistics), starting from the second dimension, qualitative aspects become increasingly relevant and require access to insider information. Such information can be collected by reviewing documents, through participant observation, including ‘online observation’ [62], as well as through interviews.

Moreover, moving from left to right, the dimensions grow increasingly complex to evaluate. ‘Expansion of knowledge’, for instance, should provide an ideal measure of the effectiveness of the CoP^KM^ strategy. However, its measurement poses important methodological challenges. Firstly, the distribution of knowledge may not be even among members. Secondly, the intangibility of some types of knowledge makes measurement complex. Additionally, in order to assess ‘expansion’, observations at two points in time, at least, are required. Finally, there may be problems with the attribution of observed changes to the CoP^KM^. Similarly, focusing on the following dimension (‘more knowledge-based policies and practices’) would be critical to capture the ‘evidence-policy’ gap. However, it would be complex to isolate the contribution of the CoP^KM^ from other factors. Indeed, changes in policy and practice are often incremental and many factors contribute to policy change (such as the political economy context, the balance of powers, windows of opportunity [18,63], as well as other knowledge strategies in place, e.g., the production of policy briefs by researchers). Qualitative techniques could be developed or adapted (see, for example, ‘contribution mapping’ proposed to assess the role of research [64]) to capture this dimension. The last dimension (‘better health and welfare outcomes’) is extremely difficult to assess. In this case, the adoption of qualitative evidence, for example in the form of reports by key informants on how the CoP^KM^ was able to influence policies that in turn could contribute to better health outcomes, may be more useful than applying quantitative methods or prospective research.

Additional File 1 proposes a list of indicators and questions with reference to each element of the framework. It is important to note that these indicators are provided only to illustrate and clarify the theoretical issues, and should be carefully adapted for any application of the conceptual framework.

### A first step towards empirical research

It is obviously premature to assess whether the proposed framework fits the many various needs of the different stakeholders of any CoP^KM^. As mentioned earlier, its development has rested on two sources: the literature and the experience of the authors. The former has not been sufficiently structured up to this point. The latter is context-bound and, at this stage, derived from a limited number of CoP^KM^; this is a limitation. Validation will depend on the emergence of an ambitious evaluation agenda on the CoP^KM^ active in health policy, which will depend itself on the consolidation of a broad commitment towards collaborative models of knowledge management across actors in health policy.

Transnational CoP^KM^ in health policy have recently witnessed growth. New communities are being established and membership is increasing daily. Our team’s research plan envisages operationalizing and testing the conceptual framework in the near future to respond, at two levels, to the evaluation needs of the Harmonization for Health in Africa initiative’s CoP^KM^, with which we collaborate. On the one hand, facilitators need to perform a self-assessment by continuously monitoring and documenting the development of their community to identify success factors and best practices in order to improve its effectiveness. On the other hand, CoP^KM^ are a research topic in their own right. Within the FEMHealth project^e^, the Financial Access to Health Services CoP will be under external scientific scrutiny by an anthropological researcher, looking at its effectiveness as a dissemination strategy and as a tool to transform knowledge and expertise into policy-related information. Other lines of research may emerge in the future and we encourage other CoP^KM^ to conduct assessments under the proposed framework. The advantage of such a research agenda lies in adopting a common framework for analysis and assessment of transnational CoP^KM^ in health policy, which would enable learning across communities.

## Conclusions

Effective knowledge management processes are widely recognized as fundamental to improve policy and health systems. Advocates of CoP^KM^ believe that they can be a key strategy to bridge evidence, policy-making and implementation by linking all actors of the system and creating a platform through which they transfer implicit and explicit knowledge, coordinate and collaborate towards the common purpose. Monitoring, analysing and assessing these communities, as well as understanding the determinants of their success, is of importance in order to respond to the challenge of building more effective and equitable health systems for all.

This paper represents a first step in the development of an evaluation and research agenda. Empirical research encompassing both self-evaluations and external assessment will be essential to provide further information on the effectiveness of CoPs as a knowledge management strategy in health policy.

## Endnotes

^a^For example, the CoPs of the Global Health Delivery Online platform (http://ghdonline.org/), the Emerging Voices project (http://www.ev4gh.net/), the Communities and Discussion Forums of the Implementing Best Practices in Reproductive Health Knowledge Gateway (http://www.knowledge-gateway.org), as well as the CoPs of Health Space Asia (http://healthspace.asia/) and those launched under the Harmonization for Health in Africa initiative (http://www.hha-online.org/hso).

^b^This paper is part of a larger theoretical and empirical agenda of work and research pursued by our team. In particular, the authors are involved in several communities of practice of the Harmonizing Health in Africa initiative, supported by the African Development Bank, UNAIDS, UNFPA, UNICEF, USAID, WHO, the World Bank, France, Japan and Norway to provide regional support to governments in Africa in strengthening their health systems. Our group is more particularly committed to four communities of practice focusing on Performance Based Financing, Financial Access to Health Services, Evidence-Based Planning and Budgeting, and Health Service Delivery. More information about Harmonization for Health in Africa can be found at http://www.hha-online.org/hso.

^c^One could easily apply this agenda to some phenomena in global health. A good candidate would be, for instance, the community of scholars committed to ‘systematic reviews for evidence-based health policy’. They clearly share a domain of interest, are concerned with improving their repertoire of practices and have developed a strong identity. Research could investigate the mechanisms they set up to govern their agenda, the issue of internal power, their relationship with the rest of the scientific community, the risk of dogmatism, and so on.

^d^In fact, using a label ‘abusively’ (consciously or not) is a standard social practice: doing so can help in ‘marketing’ the endeavour, by providing legitimacy or sounding ‘new and innovative’. The most important is to keep in mind that there are CoP^KM^ which fail to match most of the attributes of a CoP (and possibly struggle because of that) and that they are collaborative arrangements (e.g., networks) not named ‘community of practice of something’ but actually are CoPs.

^e^FEMHealth is a European Union-funded research program launched in January 2011, which focuses on fee exemption policies for maternal healthcare in Burkina Faso, Benin, Mali and Morocco. The Work Package 5 of the project relates to the dissemination strategy of the main findings and it will adopt a CoP as an innovative approach for it. The CoP itself will be evaluated. More information on FEMHealth is available at http://www.abdn.ac.uk/femhealth/.

## Abbreviations

CoP: Communities of Practice; CoPKM: Communities of Practice with knowledge management; ICT: Information and communication technologies; PBF: Performance-based financing.

## Competing interests

The authors declare that they have no competing interests. For their work as facilitators of CoPs, BM receives funding from the African Development Bank (ADB) and AK from UNICEF.

## Authors’ contributions

All authors participated in the identification of the research question and the conception of the study design. MPB and BM carried out the literature review and drafted a first version of the conceptual framework. All authors suggested further literature to review and commented extensively on the conceptual framework at various stages of its development. MPB, BM, DH, AK, YK, IL, and IS participated in the consultation meeting in Antwerp (August 2011). BM, DH, AK, YK, IL, IS, SW participated in the consultation meeting in Bamako (November 2011). All authors read and approved the final manuscript.

## Supplementary Material

Additional file 1 Indicators and questions relevant for each of the elements of the conceptual framework. Click here for file
