# Supplementary material for: Assessing communities of practice in health policy: a conceptual framework as a first step towards empirical research
Source: Health Res Policy Syst. 2013 Oct 20;11:39. doi: 10.1186/1478-4505-11-39 (PMC3819672; doi:10.1186/1478-4505-11-39)
Supplement: Additional file 1 — Indicators and questions relevant for each of the elements of the conceptual framework. [file 1478-4505-11-39-S1.docx]

**Additional file 1**: Indicators and questions relevant for each of the elements of the conceptual framework

*Note that indicators are not meant to provide a “measure” of good or bad performance of CoP^KM^, but they aim to provide a series of issues and practical questions, in order to facilitate analysis. As explained in the paper, depending on the specific needs of the exercise, only some indicators among these dimensions and elements will be the identified as more relevant for the analysis and evaluation of specific CoPs.*

| **Elements** | **Sub-elements** | **Indicators** |
| --- | --- | --- |
| **AVAILABLE RESOURCES (ideally measured at different stages of the CoP^KM^ development)** | | |
| Knowledge resources | Expertise of the members | - Demographics of members: number and detailed profile of members (skills, ‘niche’, ‘know-how’, years of experience in the domain, etc.) - Number, % and characteristics of active members - Coverage achieved by the CoP^KM^ (proportion of the experts in the domain members of the community) |
|  | Access to information | - Type of information the CoP members have access to (subscription to scholarly journals, internet access, libraries, etc.) and ease of access |
| Time resources | Time spent by members on CoP activities | - Time spent on CoP^KM^ activities by members (% of total working time) |
|  | CoP employees | - Number of people employed for the CoP^KM^ (full or part-time) |
| Financial resources | Budget | - Funding: amount, predictability, fungibility - In-kind allowances to the CoP (meeting space, web space, materials, etc.): value, predictability |
|  | Role of sponsors | - Number and identity of sponsors - Other roles of sponsors in the health policy process (internationally, regionally or nationally) |
| Political resources | Buy-in of key organizations | - Support and participation by organizations influential in the specific domain of knowledge |
|  | Formal recognition | - Quotes from CoP^KM^ outputs/activities in journals and official documents |
|  | Informal recognition/reputation | - Feedback from stakeholders (inside and outside of the community) - Mention of the CoP^KM^ in websites, blogs, conferences, other discussions - Links to the CoP on other websites |
| Technological resources | ICT tools | - Type of ICT tools used by the CoP^KM^ and reasons for their selection (mapping can be done along categories: asynchronous/synchronous; individual participation/community cultivation – Wenger et al (42)) - Synergetic approach to ICT channels - Level of internet connectivity of members in different areas and proportion of members actively using ICT tools - ICT skills of participants - Availability of ICT coaching to members (a person/team or specific support activities) |
| **Elements** | **Sub-elements** | **Indicators** |
| **STRATEGIES TO MOBILIZE RESOURCES (ideally measured at different periods of the CoP^KM^ development)** | | |
| Define the focus, promote, assess and make visible the CoP  🡪 mobilize financial and political resources | Objectives | - Clear definition of the CoP^KM^ domain, objectives and mission. When was it defined? Where (in documents, orally,…)? - Awareness of CoP^KM^ objectives among members - Alignment of CoP^KM^ activities on its objectives, especially in terms of improving the repertoire of practices. |
|  | Activities | - Monitoring and reporting of activities to all stakeholders (incl. members and sponsors). - Accountability of the CoP^KM^ to members and sponsors. |
|  | Budget | - Annual budget produced and reviewed regularly. - CoP’s capacity to generate additional funds. - Number and profile of people/team in charge of ensuring and mobilizing the CoP funding. |
|  | (Self)evaluation | - Regular CoP^KM^ evaluations and type of evaluations (internal vs. external). - Feedback of evaluation’s results to members. - Adoption of recommendation and evaluations results to change the practice of the community. |
|  | Reflection | - Frequency and quality of meta-conversations about the CoP^KM^. |
| Cultivate the community dimension  🡪 mobilize knowledge and time resources | Power issues | - Role or different actors, groups, niches or agencies (e.g., equality of prominence of some over others). - Role of sponsors in influencing the discussion. - Way issues are brought up and discussions started. |
|  | Hierarchy and participation | - Perceptions of members about the possibility of usefully and equally contributing and that their contribution is valued. - Type of participation by members: as individuals, as agencies’ representatives, etc. |
|  | Regulatory mechanisms | - Existence and use of (written or non-written) codes to avoid inappropriate contributions and misbehavior. - Perceptions of members on the regulation of the contributions |
|  | Trust | - Number of job ads shared - Number of posts that are “personal” - Number of referrals or recommendations - Confidence in bringing up problems/failures and asking for advice on ongoing work - Self-reported collaborative spirit - Self-reported levels of trust (across three dimensions: perceived competence, integrity and benevolence of the CoP^KM^ - Usoro et al (48) |
|  | Fluidity of the community | - Language (% of messages in one language; % of messages in all languages; perception of members) - Culture (presence of cultural differences in the approach to the CoP, and existence of potential cultural misunderstandings or barriers) - Virtual contacts vs. contacts in person/real life - New contacts made through the CoP - Number and success of in-person activities vs. online activities - Number of interactions between members outside of CoP activities |
|  | Ownership/ identity  Sense of belonging | - Number of congratulations/condolences/etc. notes - Informal meetings, dinners, lunch (outside formal CoP activities) - Perception of members on their sharing a common identity. Ways this identity is created/defined. |
|  | Confidence | - Perception of members about being empowered by their belonging to the CoP^KM^. |
| Aligning CoP’s activities to individual and organizational expectations of benefit  🡪 mobilize knowledge and time resources | Value and benefits of participation | - Correlation between use of resources and participation - Reasons for becoming a member of the CoP^KM^, for individuals - Reasons for maintaining active participation to the CoP^KM^ (for individuals) (question of ‘lurkers’ vs. active participants, and on how to maintain active participation on a voluntary basis) - Reasons for participations for organizations - Personal benefits (see: expansion of knowledge) - Organizational benefits (see: expansion of knowledge) |
|  | Cost of participation | - People unsubscribing (number and profile) - People no longer active - Organizations withdrawing their support or their employers’ time - Reasons for not reading messages from other members - Reasons for unsubscribing - Reasons for not attending face-to-face events - Personal and organizational costs |
| Use of ICT in an effective and cost-effective manner  🡪 mobilize technological resources | User-friendliness | - Use of ICT tools that are supported by members’ hardware/software. - Provision of ICT support (posts on ITC questions, coach, activities, …). - Perceived user-friendliness of the community tools by members. |
|  | “Landscape of CoP activities” (Wenger et al 2005) | - Number and % of non-ICT based events vs. ICT-based tools/activities - Number of participants to online tools vs. other events - Geographical distribution of participants to different events/tools/activities |
|  | Adoption of good practices to foster online activities | - Check-list of recommended design features - Kraut & Resnick (51) |
|  | Cost effectiveness of ICT | - Cost of tools/software adopted (costly, free/open sources, …). Cost-effectiveness of these tools. - Presence of ICT steward required to manage the technical side of the CoP^KM^. Cost-effectiveness of this investment. |
| **Elements** | **Sub-elements** | **Indicators** |
| **KNOWLEDGE MANAGEMENT PROCESSES (ideally, measured across members belonging to the different knowledge ‘niches’)** | | |
| KM processes (activities and interactions) | Level of activity | - Number of meetings, workshops, other activities (both in person and online, formal and informal, between a small group or involving the entire community).   For example:   - Web page visits - Number of posts/queries - Number of new discussions - Number and timeliness of responses - Possibilities of personal interactions and networking |
|  | Quality of interactions | - Adoption of good practices in knowledge management in the facilitation processes on online fora and in face-to-face events - Quality and usefulness of responses/debates/activities/interactions (subjective and objective evaluation: anecdotes on useful tips, thank you notes/kudos files, user rankings, expert evaluation, citations by others) - Responsiveness of interactions (i.e., rapidity of CoP reaction to new information, brought by by media, or particular event/crisis, etc.) |
|  | Relevance of activities / Level of engagement | - Alignment of activities on the domain of interest - Alignment of activities on the improvement of the repertoire of practices - % of members using various resources - % of members using various communication tools - Average number of members involved in a discussion - Length of threads - Relevance of activities for the organizations that participate or allow their employees to participate. - Relevance of activities to the CoP^KM^’s objectives and aims. |
| **Elements** | **Sub-elements** | **Indicators** |
| **EXPANSION OF KNOWLEDGE (expansion measurement required comparisons at different points of time and across individuals)** | | |
| Type of knowledge | Explicit or implicit | - Means and tools of knowledge share and production, in particular for implicit/tacit knowledge (for example, by means of travel of members for consulting assignments, by trainings or study tours, or by other activities). - Prevalence of one type of knowledge than the other. Reasons and consequences. |
|  | More or less synthesized | - Regular production of summaries of events and discussions (number of synthesis posts) - Archives - Systematic and easy-to-search databases - Types of documents: report, studies, guidelines, … |
|  | Scientific evidence, field experience, experts’ opinions | - Ways of producing and sharing scientific knowledge. - Role of scientific evidence vs. expertise and field experience. - Prevalence of one type of knowledge than the other. Reasons and consequences. |
|  | Knowledge is harmonized and accepted between different ‘niches’ | - Number of joint projects - Number of co-authored documents - Collaborative spirit - % of participants from different ‘regimes’ - % of active participants from different ‘regimes’ and their role - Contribution of CoP in making knowledge holders closer. Examples. - Structural patterns in the social interactions (e.g., using Social Network Analysis) - Interactions between ‘regimes’ (examples). |
|  | Debated or consensual | - Number of “debate” posts/activities. - Reaction to critical issues. Perception of members on posting about critical issues. - Ability and interest of the community of reaching a consensus, rather than leaving controversial issues unanswered. |
| Potential value | Individual level | - Main beneficiaries of knowledge expansion - Skills/competences acquired (personal benefit) - Increased speed and accuracy of work (self-reported and externally evaluated, for ex. by managers) - Information received - Changes in perspective - New contacts made |
|  | Organizational level | - Number of outputs (documents, databases, summaries, etc.) produced - Frequency of downloads - Quality of outputs (perceived and objective) - Coverage of relevant topics - Higher level of technical capacity |
| Applied value | Use of CoP tools and documents | - Number of contacts in database, archive, etc. - Frequency of downloads - Number of citations of CoP^KM^ outputs in papers, articles and documents |
|  | Actual implementation of CoP advice/best practices | - Reported number of problems solved - Reported number of lessons adopted - Anecdotes/stories on how and why CoP was useful |
| **Elements** | **Sub-elements** | **Indicators** |
| **KNOWLEDGE-BASED POLICY DECISIONS AND PRACTICES** | | |
| Legitimacy of knowledge |  | - Type of evidence mostly used as an argument in discussions. - Reporting and validation of experiences/field practices of members. - Ways of using “scientific” evidence. - Opinions: presented as such, discussed, validated? - Perception of actors (from different ‘niches’) about relevance and legitimacy of knowledge produced and shared. |
| Acceptance of innovation | Challenges and new ideas | - Number of posts/activities from non-members - Number of posts presenting a critique/challenge to the group - Posts challenging assumptions and reactions |
|  | Community openness | - Changes in perspective (and their documentation). - Community membership turn-over. Number of new members per month. - Time taken by a new member to become an active participant. - Participants perception about openness of the community to debate, new ideas and new members - “Are we the truth-holders?” |
| Influence policy and practice |  | - Development of new criteria/outcome measures in the field of interest. - Contribution of the CoP^KM^ work in changing the way of understanding the field of interest. - Contribution of CoP^KM^ in reaching a consensus. - Capacity to influence policy (difficult to assess, see for ex: “policy impact database”) - Capacity to influence implementation and practices |
| **Elements** | **Sub-elements** | **Indicators** |
| **BETTER HEALTH AND WELFARE OUTCOMES** | | |
| Health outcomes |  | - Changes in health outcomes among the population. - Role of the CoP^KM^ for these changes. - Ways by which the CoP^KM^ influenced this outcome. |
